# Immunology of IL-12: An update on functional activities and implications for disease

**DOI:** 10.17179/excli2020-3104

**Published:** 2020-12-11

**Authors:** Karen A.-M. Ullrich, Lisa Lou Schulze, Eva-Maria Paap, Tanja M. Müller, Markus F. Neurath, Sebastian Zundler

**Affiliations:** 1 Department of Medicine and Deutsches Zentrum Immuntherapie, University Hospital Erlangen, Friedrich-Alexander-University Erlangen-Nuremberg, Germany

**Keywords:** IL-12, TH1 cells, STAT4, immunology, cytokines, ustekinumab

## Abstract

As its first identified member, Interleukin-12 (IL-12) named a whole family of cytokines. In response to pathogens, the heterodimeric protein, consisting of the two subunits p35 and p40, is secreted by phagocytic cells. Binding of IL-12 to the IL-12 receptor (IL-12R) on T and natural killer (NK) cells leads to signaling via signal transducer and activator of transcription 4 (STAT4) and subsequent interferon *gamma* (IFN-γ) production and secretion. Signaling downstream of IFN-γ includes activation of T-box transcription factor TBX21 (Tbet) and induces pro-inflammatory functions of T helper 1 (T_H_1) cells, thereby linking innate and adaptive immune responses. Initial views on the role of IL-12 and clinical efforts to translate them into therapeutic approaches had to be re-interpreted following the discovery of other members of the IL-12 family, such as IL-23, sharing a subunit with IL-12. However, the importance of IL-12 with regard to immune processes in the context of infection and (auto-) inflammation is still beyond doubt. In this review, we will provide an update on functional activities of IL-12 and their implications for disease. We will begin with a summary on structure and function of the cytokine itself as well as its receptor and outline the signal transduction and the transcriptional regulation of IL-12 secretion. In the second part of the review, we will depict the involvement of IL-12 in immune-mediated diseases and relevant experimental disease models, while also providing an outlook on potential translational approaches.

## Introduction

Dozens of cytokines have meanwhile been described and the knowledge on the stimuli that induce their release, the signaling they trigger and the cellular responses they cause has substantially increased our understanding of immune processes in the organism. Cytokines are regarded as crucial para- or autocrine mediators of inter-cellular communication regulating functions like proliferation, differentiation and maturation (Neurath, 2014[138]). 

One important group of cytokines are interleukins, in which the twelfth to be described in 1989 was Interleukin-12 (IL-12). In more than three decades, numerous papers have reported effects and functions of IL-12. Here, we will briefly point out the biochemistry of IL-12 before reviewing its involvement in immune-mediated pathologies.

## Interleukin-12

Two groups independently described IL-12: Kobayashi et al. reported the identification of the natural killer cell stimulating factor (NKSF) in 1989 (Kobayashi et al., 1989[95]) and Stern et al. discovered the cytotoxic lymphocyte maturation factor (CLMF) in 1990 (Stern et al., 1990[174]). Soon thereafter, CLMF and NKSF were found to be identical (Gubler et al., 1991[71]) and the name IL-12 was proposed.

IL-12 consists of two subunits, which are connected by disulphide-bonds (Kobayashi et al., 1989[95]; Stern et al., 1990[174]). The smaller p35 monomer (35 kDa α-chain) is encoded on chromosome 3, while the gene for the larger p40 monomer (40 kDa β-chain) is located on chromosome 5 (Sieburth et al., 1992[171]). Co-expression results in the formation of the biologically active p70 heterodimer (Gubler et al., 1991[71]).

**Figure 1:**
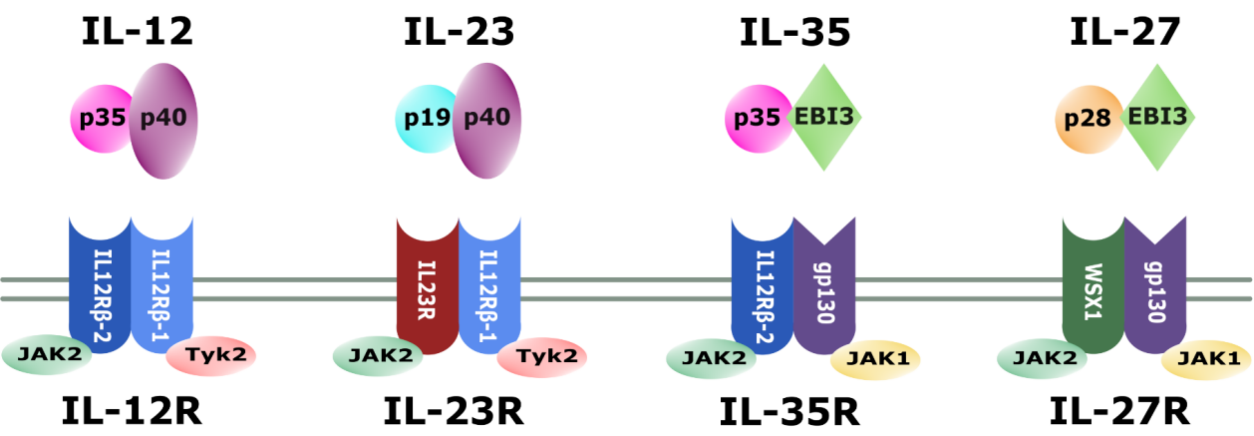
The IL-12 family. Schematic representation of IL-12 family members, their associated receptors and corresponding subunits

Within the 'IL-12 family' of cytokines, monomers combine with different partners to create various cytokines. While p35 may also pair with Epstein-Barr virus induced gene 3 (EBI3) to yield IL-35 (Niedbala et al., 2007[141]), the p40 subunit in combination with the p19 monomer leads to the formation of IL-23 (Lupardus and Garcia, 2008[113]). The fourth member of the family is IL-27, which is composed of EBI3 and the p28 subunit (Vignali and Kuchroo, 2012[196]) (Figure 1(Fig. 1)).

Due to the lower expression of the IL-12 α-chain compared to the β-chain, only free β-chains, p40 homodimers or the heterodimer are secreted (D'Andrea et al., 1992[42]). Structurally, the p40 subunit shares some features with the IL-6 receptor, whereas the p35 subunit is similar to the granulocyte colony-stimulating factor (G-CSF) and IL-6 (Gubler et al., 1991[71]). It has been demonstrated in mice that p40 homodimers regulate the activity of IL-12 by counteracting IL-12-induced signaling via competition with IL-12p70 for binding to the receptor (Gately et al., 1996[59]). Further functions of p40 homodimers have been described, e.g. roles in the migration of dendritic cells (DCs), allograft rejection or chemotactic activity with regard to macrophages (Cooper and Khader, 2007[39]; Ha et al., 1999[73]). 

## The Interleukin-12 Receptor

Reflecting the heterodimeric structure of the cytokines of the IL-12 family, the corresponding receptors also consist of two subunits. IL12-receptor β1 (IL-12Rβ1) is encoded on chromosome 19 and has a molecular weight of 100 kDa. It is a transmembrane protein with the extracellular domain consisting of 516 amino acids that is responsible for the interaction with IL-12p40 (Chua et al., 1994[36]; Presky et al., 1996[156]). Consistently, it is also part of the receptor for IL-23, where it pairs with IL-23R (Parham et al., 2002[151]). The gene for IL-12Rβ2 is located on chromosome 1 and is translated to a 130 kDa transmembrane protein, with 595 amino acids forming the extracellular domain. Signal transduction into the cell derives from IL-12Rβ2, which interacts with IL-12p35 (Presky et al., 1996[156]; Zou et al., 1997[224]) and is, thus, in combination with glycoprotein 130 (gp130), also part of the IL-35 receptor (Collison et al., 2012[38]). The IL-27 receptor as the fourth receptor of the family is composed of gp130 together with the interleukin 27 receptor subunit alpha (WSX1) (Pflanz et al., 2004[152]) (Figure 1(Fig. 1)).

Since NK cells and T cells are the main targets of IL-12, the expression of IL-12R is predominantly confined to these cell types (Desai et al., 1992[45]). In particular, antigen contact of naïve T cells induces upregulation of IL-12Rβ2, which is subsequently maintained by interferon *gamma* (IFN-γ) signaling, but may be counteracted by IL-4 (Szabo et al., 1997[175]). This hints at a vital role for the commitment of T cells to different effector T (T_eff_) cell lineages such as cells with a T helper type 1 (T_H_1), but not a T_H_2 phenotype and, consistently, only the former cells express IL-12Rβ2.

## Regulation of IL-12 Secretion

IL-12 is primarily produced by professional antigen-presenting cells (APCs) such as B cells and DCs as well as phagocytes including monocytes, macrophages and granulocytes (Hsieh et al., 1993[81]; Heufler et al., 1996[77]; Macatonia et al., 1993[116]). 

While the production of IL-12p35 is predominantly regulated at the translational level, transcriptional regulation is responsible for the amount of IL-12p40 expressed. The initial signal triggering IL-12 expression is the exposure of the above mentioned cells to bacteria, viruses, fungi or parasites. Pathogen associated molecular patterns (PAMPs) such as lipopolysaccharide (LPS) or CpG DNA expressed or contained in such commensals or pathogens are recognized by pattern recognition receptors (PRRs) of the toll like receptor (TLR) family. This leads to the activation of several transcription factors regulating IL-12 production, most importantly NF-κB and interferon regulatory elements (IRFs) (Goriely et al., 2008[65]). 

Due to the different chromosomal locations (Liu et al., 2003[109]) and as mentioned above, there are important differences in the regulation of p40 and p35 production. The synthesis of the p40 chain greatly exceeds the production of the p35 chain, suggesting that the synthesis of the p35 chain is the rate-limiting step of IL-12 secretion (Snijders et al., 1996[173]). Moreover, most of the TLRs are linked to the expression of IL-12p40, while expression of p35 is induced by only a limited subset of these receptors, including TLR3, 4 and 8. In addition to direct regulation of IL-12 production, activation of TLRs also leads to the secretion of IFN-β and IFN-γ, whose signaling, in turn, induces activation of IRF-1, IRF-7 and IRF-8 (Goriely et al., 2008[65]; Najar et al., 2017[135]; Gautier et al., 2005[60]). All three IRFs induce p35 and IRF-7 and IRF-8 also induce p40 (Ma et al., 2015[115]; Zhao et al., 2017[219]). 

The IL12A gene is transcribed throughout, however, the mRNA contains an inhibitory ATG codon blocking its translation. Upon TLR signaling as described above, transcription is initiated from different genomic positions. Since ATG codons are missing in the resulting mRNA, translation into IL-12p35 proceeds (Wang et al., 2000[203]). 

The IL12B gene is subject to transcriptional regulation by a number of transcription factors, most importantly NF-κB and Ets. Moreover, Spi-1, AP-1, IRF-1, erythroid Krüppel-like factor 1 (KLF-1), nuclear factor in activated T cells (NFAT) and interferon consensus sequence-binding protein (ICSBP) have been described to be involved (Ma et al., 2015[115]; Zhao et al., 2017[219]). Furthermore, some groups showed an inhibitory effect of Nuclear Receptor Subfamily 4 Group A Member 1 (NR4A1) on the expression of IL-12p40 (Murphy and Crean, 2015[131]; Ipseiz et al., 2014[85]).

In addition, IL-12 expression is regulated via interaction of APCs with T-cells through CD40 and its ligand CD40L. CD40 signaling through H-Ras and K-Ras enhances p38 mitogen-activated protein kinase (MAPK)-mediated pro-inflammatory IL-12 production (Snijders et al., 1996[173]; Nair et al., 2020[134]).

An important positive feedback loop increasing IL-12 secretion is so-called IFN-γ priming (Liu et al., 2003[109]; Ma et al., 2015[115]). IFN-γ release downstream of IL-12 further boosts IL-12 production via induction of IL-12p35 by IRF-1 and of p40 by ICSBP (Wang et al., 2000[203]; Grumont et al., 2001[70]). 

Taken together, the high degree of regulatory mechanisms involved in IL-12 secretion illustrates the complexity of the processes, in which this cytokine is involved and underscores that dysregulation might be a switchpoint for disease development. 

## IL-12 Signal Transduction

**Figure 2:**
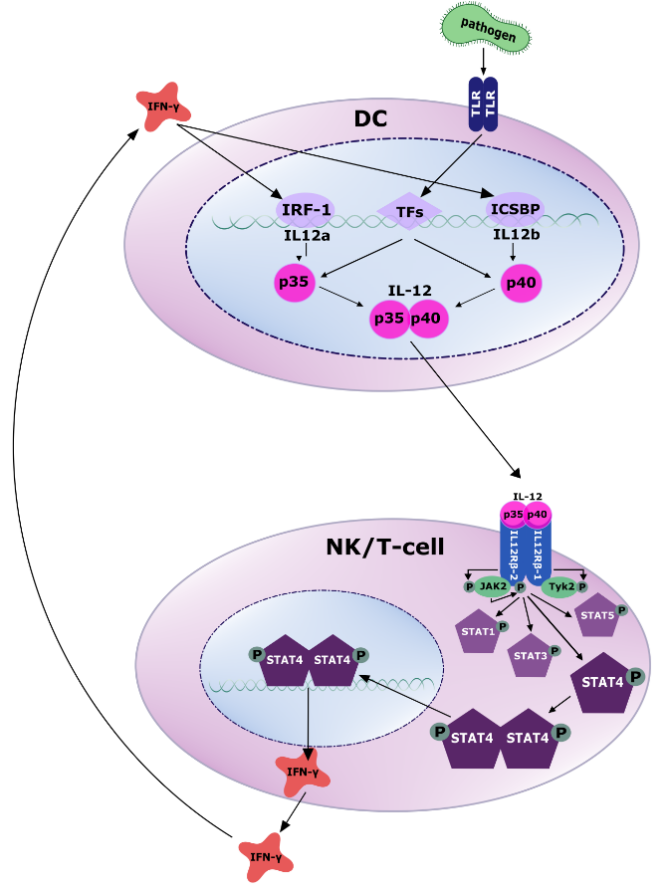
Secretion and signaling of IL-12. Antigen-presenting cells (APCs) like dendritic cells sense PAMPs (pathogen-associated molecular patterns) through toll like receptors (TLRs). Subsequently, several transcription factors are activated to induce the transcription of IL-12p35 and IL-12p40 (for more details confer text). The secreted IL-12 heterodimer binds to its receptor on NK and T cells, recruits the tyrosine kinases JAK2 and TYK2 and activates JAK2 by tyrosine phosphorylation. Activated JAK2 phosphorylates the IL12Rβ2 subunit, which in turn activates STAT4 via phosphorylation. Subsequently, phosphorylated STAT4 homo- or heterodimerizes, enabling translocation to the nucleus, where it regulates gene transcription by binding to target DNA. A main target gene is IFN-γ, which in turn induces transcriptional activation of IL-12 production via IRF-1 and ICSBP.

Binding of the two IL-12 subunits to the two chains of the IL-12 receptor, IL-12Rβ1 and IL-12Rβ2, activates the Janus kinase (JAK)-signal transducer and activator of transcription (STAT) pathway of signal transduction. Specifically, IL‐12Rβ-1 subsequently recruits the JAK family member tyrosine kinase 2 (TYK2), whereas IL-12Rβ2 associates with JAK2, resulting in phosphorylation of JAK2 (Bacon et al., 1995[10]; Zou et al., 1997[224]). This activates the kinase activity of JAK2, which now, *vice versa*, phosphorylates a tyrosine residue of the associated receptor subunit. STAT molecules contain SRC homology domains (SH2), which, in a next step, bind to phospho-IL-12Rβ2 exposing the STATs to JAK and leading to their phosphorylation. Association of these activated transcription factors to homo- or heterodimers enables subsequent nuclear translocation. By binding to specific DNA sequences, they promote or repress gene transcription (Naeger et al., 1999[133]; Zhong et al., 1994[221]; Xu et al., 1996[208]; Jacobson et al., 1995[86]; Lamb et al., 1996[104]) (Figure 2(Fig. 2)). STAT4 is the most important downstream target of IL-12, while effects on STAT1, STAT3 and STAT5 molecules play minor roles (Trinchieri, 2003[184]). 

Moreover, IL-12R signaling activates mitogen-activated protein kinase kinase 3/6 (MKK) and p38 MAPK, which support the secretion of IFN-γ in activated T cells and T_H_1 cells. Importantly, this pathway is mediated by a STAT4-independent mechanism and correlates with increased STAT2 (Zhang and Kaplan, 2000[218]). 

## Cellular Functions of IL-12

**Figure 3:**
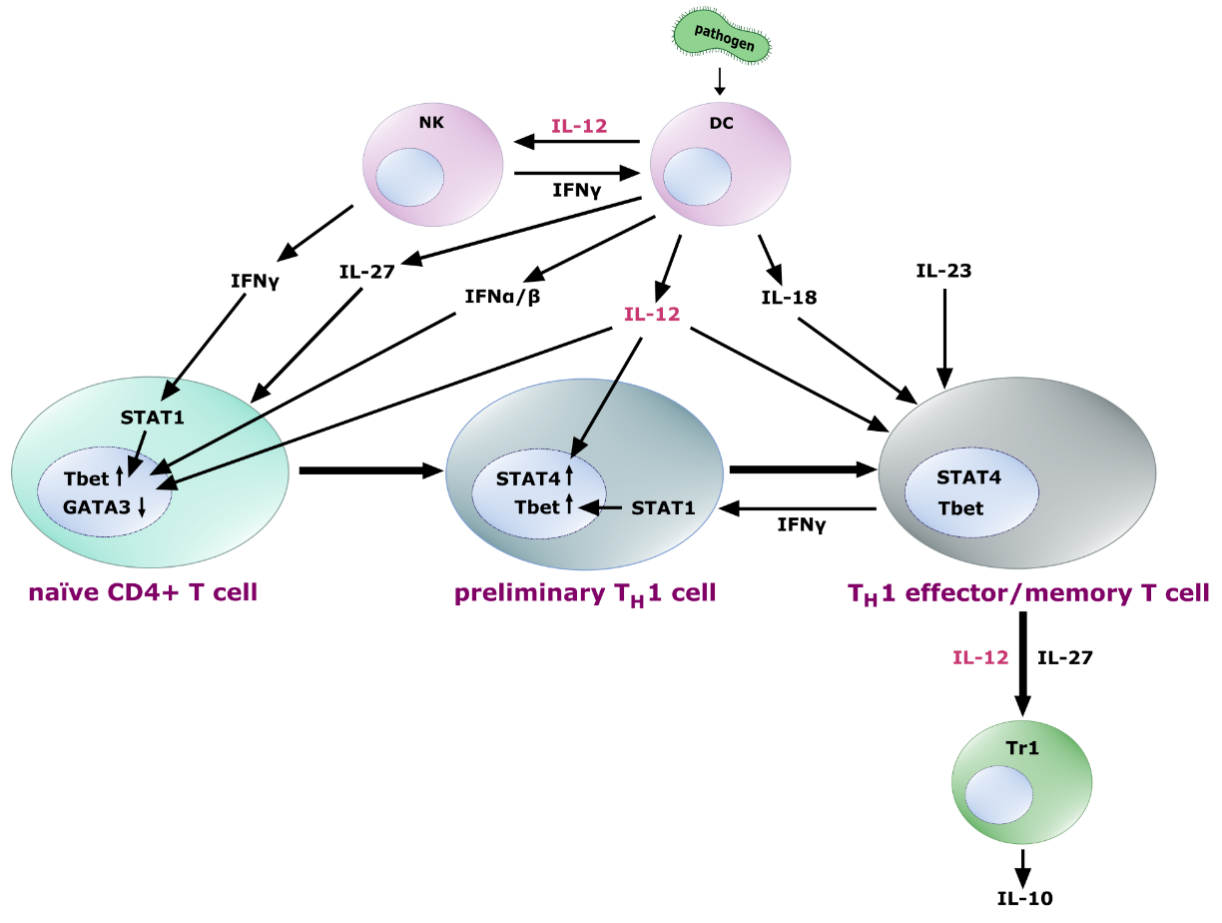
Role of IL-12 in T_H_1 differentiation. Naïve T cells exposed to IL-27 express the IL-12 receptor heterodimer, sensitizing the cells for the influence of IL-12, which - together with IFN-γ from NK cells and feedback loops, IFN-α and IFN-β induces upregulation of the transcription factor Tbet and downregulation of GATA3 leading to a preliminary T_H_1 commitment. Further exposure to IL-12 leads to the upregulation of STAT4 in these early T_H_1 cells, followed by their differentiation into T_H_1 effector and memory T cells. IL-18 and IL-23 contribute to the fixation, amplification and maintenance of the T_H_1 cell effector functions. Differentiated T_H_1 cells produce IFN-γ to enhance Tbet expression via STAT1, thus closing a positive feedback loop. Further, they are able to promote IL-10 secreting Tr1 cells, a process probably mediated by IL-12 and IL-27 signaling.

As already mentioned, a main effect of IL-12 is the induction of IFN-γ production, by which the cytokine is importantly implicated in adaptive as well as innate immune processes (Figure 3(Fig. 3)) (Trinchieri, 2003[184]; Lyakh et al., 2008[114]). 

While having no proliferative effect on resting peripheral T cells or NK cells, IL-12 directly induces proliferation of these cells in case of pre-activation. Moreover, by inducing the transcription of genes of cytotoxic granule-associated molecules, such as perforin and granzymes, and by upregulation of the expression of adhesion molecules, IL-12 enhances the generation and cytotoxic activity of cytotoxic T lymphocytes (CTLs), lymphokine-activated killer (LAK) cells and NK cells, which also secrete IFN-γ (Trinchieri, 1998[185], 2003[184]; Tait Wojno et al., 2019[177]). Even low concentrations of IL-12 promote IFN-γ production in a highly efficient way. Furthermore, IL-12 is also very important as IFN-γ-inducer in synergy with other activating stimuli. E.g., for T cells and NK cells, IL-12 acts synergistically with IL-2 to rapidly upregulate IFN-γ (Trinchieri, 2003[184]; Chan et al., 1992[33]). While the cytokine IL-18 alone is ineffective in regulating IFN-γ, in synergy with IL-12, it induces gene transcription via the transcription factors STAT4 and AP-1. This is particularly important for the induction of IFN-γ by cell types such as macrophages, DCs or B cells that are no conventional sources of this cytokine (Trinchieri, 2003[184]; Walker et al., 1999[200]; Barbulescu et al., 1998[14]). Interestingly, STAT4 is required for the production of IFN-γ downstream of IL-12 in both CD4^+^ and CD8^+^ T cells, but only CD4^+^ T cells require IL-12 and STAT4 for the production of IFN-γ following antigen recognition and T cell receptor (TCR) signaling suggesting alternative regulatory pathways in different lymphocyte subsets (Trinchieri, 2003[184]; Carter and Murphy, 1999[30]). It was also shown that IL-12 pre-treatment of CD4^+^ and CD8^+^ T cells enhances TCR-induced IFN-γ, tumor necrosis factor *alpha* (TNF-α), IL-13, IL-4 and IL-10 production and intensifies oxidative metabolism (Vacaflores et al., 2016[191], 2017[192]).

Due to and related to its predominant effect on IFN-γ transcription, IL-12 is a potent inducer of T_H_1 cell development. However, it is not sufficient to guide this process, since previous signaling downstream of IL-27 released by DCs is required in naïve T cells to make the cells “IL-12-sensitive” by expression of IL12Rβ2 (Pot et al., 2010[154]). After release of IL-12 and induction of IFN-γ, IFN-γ signaling activates STAT1, which together with IFN-α, IFN-β and IL-12 induces the T-box transcription factor 21 (TBX21/Tbet), the key transcription factor for T cell commitment to preliminary T_H_1 cells (Trinchieri, 2003[184]). For this early phase of T_H_1 polarization, some exceptions seem to exist, in which IL-12 might not be an absolute requirement and coordinated action of other pathways might compensate, if IL-12 is missing. IL-12, however, is essential for the subsequent maturation phase (Noble et al., 2001[142]) and, together with IL-18 and IL-23 fixes, amplifies and maintains a T_H_1 phenotype during clonal expansion to effector and memory T_H_1 cells (Trinchieri, 2003[184]). Furthermore, it has been shown that the expression of genes promoting the induction of IFN-γ- and IL-21-secreting T_H_1-biased T follicular helper (T_FH_1)-like cells are also dependent on IL-12 signals, especially on the expression of Bcl-6 and inducible T-cell costimulator (ICOS) (Powell et al., 2019[155]).

Additionally, it has been shown that IL-12 also primed CD4^+^ and CD8^+^ T cells to produce IL-10, when present early during clonal expansion (Gerosa et al., 1996[61]). This might result in the development of IL-10 secreting Type 1 regulatory (Tr1) cells in response to IL-12 and IL-27 (Tait Wojno et al., 2019[177]; Pot et al., 2010[154]; Wang et al., 2011[201]), which is consistent with the observation of IL-12-dependent Tr1 cell development in visceral leishmaniasis patients (Montes de Oca et al., 2016[127]). T_H_2 differentiation, to the contrary, is counteracted by IL-12, since GATA binding protein 3 (GATA3), which is indispensable for T_H_2 polarization, is repressed in CD4^+^ and CD8^+^ T cell populations upon treatment with IL-12 or *in vivo* expansion in the presence of IL-12-producing DCs (Billerbeck et al., 2014[22]).

Taken together, IL-12 is a potent cytokine to regulate the immune response in different ways. Thus, its implication in immune-mediated diseases is obvious and will be addressed in the following part.

## Relevance of IL-12 in Different Diseases and Disease Models

Consistent with its central role in orchestrating immune responses, various studies in animal models and humans confirmed that IL-12 contributes to the pathogenesis of several immune-mediated inflammatory diseases. In the following paragraphs, we outline the current knowledge on the impact of IL-12 in the context of inflammatory bowel diseases, psoriasis, diabetes mellitus, multiple sclerosis, rheumatoid arthritis, cancer, lupus erythematosus, primary biliary cholangitis and Sjögren's syndrome. 

### Inflammatory Bowel Diseases

The inflammatory bowel diseases (IBDs) Crohn's disease (CD) and ulcerative colitis (UC) have a multifactorial pathogenesis and are marked by a misdirected and dysregulated immune response, which arises due to factors such as genetic predisposition, intestinal dysbiosis and a disruption of the intestinal epithelial barrier. Experimental colitis can be observed in genetic knockout models leading to spontaneous development of colitis as well as following the administration of chemicals like oxazolon, trinitrobenzene sulfonic acid (TNBS) or dextran sodium sulfate (DSS). Further, a so called T cell transfer colitis model can be induced by transfer of colitogenic T cells to immunodeficient mice (Neurath, 2014[138]). 

T_H_1-like colonic lymphocytes have been identified as predominant in TNBS-induced colitis and anti-IL-12p40 administration was able to eliminate inflammation (Hurtubise et al., 2019[83]) as well as to re-establish tolerance towards the intestinal microbiota (Duchmann et al., 1996[47]). Fuss et al. suggested that Fas pathway activation inducing apoptosis of T_H_1 cells might be a leading mechanism (Fuss et al., 1999[55]). Corroborating these results on transcription factor level, mice adoptively receiving CD4^+ ^T cells from STAT4-transgenic mice developed pronounced colitis and STAT4-transgenic mice themselves showed higher STAT4 expression in their lamina propria lymphocytes and were more susceptible to colitis (Wirtz et al., 1999[207]). Consistently, spontaneous colitis observed in IL-10^-/-^ mice was rescued by additional knockout of IL-12R and IL-23R (Hurtubise et al., 2019[83]) as well as by administration of a neutralizing anti-IL-12 antibody (Davidson et al., 1998[43]). Blocking IL-12 was also able to avert T cell transfer-induced colitis in immunodeficient mice which received CD4^+ ^T cells from IL-10^-/-^ donors, however this was not the case for the blockade of IFN-γ (Davidson et al., 1998[43]) indicating that IFN-γ-independent pathways are involved. In conclusion, T_H_1-dependent colitis models seem to be clearly dependent on IL-12, but IFN-γ as a major cytokine of T_H_1 cells seemed to be non-essential. An explanation for this redundancy might be that many of these studies focused on IL12p40 at a time, when IL-23 and its effects on IL-17-producing T_H_17 cells were not yet described (Moschen et al., 2019[129]).

Following the identification of IL-23, intensive research tried to dissect the mechanisms exerted by IL-12 and IL-23 on their own, although some aspects cannot finally be separated. In murine models, IL-23 was, like IL-12, able to induce STAT4 activation and overexpression was followed by colitis. Furthermore, p40 and IL-23 induction occurred subsequent to bacteria intake in DCs in the terminal ileum (Becker et al., 2003[18]). In studies focusing on the differences between IL-12 and IL-23, depletion of p19, but not p35 in IL-10^-/-^ mice led to protection from colitis development (Yen et al., 2006[213]). Consistently, while treatment with anti-p40 antibodies protected from systemic inflammatory disease and experimental colitis, treatment with anti-p19 alleviated intestinal but did not affect systemic pathology, suggesting a contribution of IL-23 to local inflammation and an IL-12-regulated systemic inflammation (Uhlig et al., 2006[189]). In TNBS colitis, to the contrary, p19 knockout was not protective due to upregulation of IL-12 suggesting that both cytokines might interact in the pathogenesis of colitis (Becker et al., 2006[17]). A recent study building on a genetically induced intestinal barrier impairment phenotype added further complexity by showing that inflammation in this model was mediated by IL-12 and IL-23 in a temporally distinct, biphasic manner. While inflammation in the early stages was driven by IL-12, the inflammatory response shifted towards IL-23 in older mice (Eftychi et al., 2019[48]). This is consistent with the assumption of some experts that the early stages of CD pathogenesis are driven by T_H_1 activation mediated by IL-12 and the following IFN-γ signaling cascade, whereas the continuation of disease is more likely driven by IL-23 (Becker et al., 2005[19]). What is further complicating a clear separation of IL-12- and IL-23-induced functions is T cell plasticity. It has been shown that IL-12 exposure shifted the phenotype of T_H_17 effector memory cells from the mesenteric lymph nodes of CD as well as UC patients towards a T_H_1-like profile suggesting that T_H_17 plasticity is taking place at inductive sites before T cell homing to gut tissues (Bsat et al., 2019[26]). 

Taken together, undoubtedly, IL-12 as well as IL-23 are implicated in the pathogenesis of IBD. Hence, they are promising targets for IBD therapy, which has further been supported by observations in IBD patients. IL-12 transcription is increased in both subtypes of IBD (Nemeth et al., 2017[137]). Additionally, CD is marked by high levels of IFN-γ produced by lamina propria lymphocytes (Fuss et al., 1996[56]) substantiating the view that CD seems to be a partly T_H_1-driven disease. Moreover, it was shown that the expression patterns of the IL-12A and IL-12B genes differed between flare-ups and remission phases and might therefore be suitable biomarkers of different disease phases (Norouzinia et al., 2018[144]). 

All these observations led to efforts to translate anti-IL-12/IL-23 strategies into clinical practice. Ustekinumab is a neutralizing p40 antibody. While early clinical studies suggested effects in CD patients with previous anti-TNF treatment, phase III trials completed in recent years have demonstrated broad efficacy and safety in inducing and maintaining remission in CD and recently also in UC (Sands et al., 2019[164]; Feagan et al., 2016[51]; Sandborn et al., 2012[162]; Adedokun et al., 2018[1]; Hanauer et al., 2020[75]). Moreover, a number of anti-p19 antibodies blocking IL-23 (e.g., risankizumab, guselkumab) are currently in phase III trials following promising data in phase II (Sandborn et al., 2020[161]; Sands et al., 2017[163]). Once finished, they will further help to understand the specific contribution of IL-12 and IL-23 to disease pathogenesis. 

### Multiple sclerosis

Multiple sclerosis (MS) is considered as a T_H_1-, T_H_17-, B- and innate immune cell-mediated disorder of the central nervous system (CNS), in which immune cell infiltration to the CNS leads to inflammatory lesions, axonal demyelination and enhanced production of pro-inflammatory cytokines (von Essen et al., 2019[198]; Segal et al., 1998[167]). Early studies focussing on p40 postulated IL-12 as a critical pro-inflammatory cytokine for MS (Adorini, 1999[2]; Karp et al., 2000[92]). Consistently, during the acute paralytic phase of experimental autoimmune encephalomyelitis (EAE), a common animal model for MS, mice showed increased expression of IL-12p40 mRNA in brain and spinal cord as well as in spleen, lymph node and liver. Furthermore, *in vivo* administration of recombinant IL-12 led to higher IFN-γ production and inflammation, which was decreased by treatment with anti-IL-12 antibodies (Bright et al., 1998[24]; Vandenbroeck et al., 2004[195]). However, later studies showed that p35^-/-^ animals were not protected from EAE, whereas p40^-/-^ mice were resistant to disease development (Becher et al., 2002[16]; Gran et al., 2002[67]). Thus, these effects could not be attributed to IL-12p70, but later be explained by IL-23 and protection of p19^-/-^ mice in the EAE model (Gran et al., 2004[66]). 

However, also here, the picture is not completely clear, since it was shown with an adoptive transfer strategy that IL-12-modulated T_H_1 cells may induce EAE via an IL-23-independent pathway, while IL-23-modulated T_H_17 cells may induce EAE through an IL-12-independent way. This indicates, that clinically similar forms of EAE may be mediated by distinct autoreactive T cell subsets pointing at synergistic or alternative functions of these parallel inflammatory pathways (Grifka-Walk et al., 2015[69]; Kroenke et al., 2008[99]). 

Recently, a huge amount of single-nucleotide polymorphisms (SNPs) with suggestive evidence of association with MS were identified by meta-analyses. These include, among others, loci which are related to IL-12 and the IL-12 family, e.g. IL-12A (rs4680534), or their downstream signaling, e.g. JAK1 (rs72922276), STAT4 (rs6738544) and TYK2 (rs34536443) (von Essen et al., 2019[198]; Ban et al., 2009[13]; IMSGC, 2010[84]). Moreover, significantly elevated levels of IL-12 were found in sera of MS patients and in cultured peripheral blood mononuclear cells (PBMCs), which were even higher in chronic progressive compared to relapsing-remitting MS (Musabak et al., 2011[132]; Balashov et al., 1997[12]). Moreover, an indirect effect of IL-12 on the pathogenesis of MS was postulated: IL-12p70 and p35 subunit, but not p40, led to increased expression of IL-7 in the CNS, which is associated with MS and EAE (Jana et al., 2014[87]).

Also, considerably enhanced expression of adhesion molecules involved in leukocyte recruitment to the CNS like C-C-motif-chemokine-receptor 5 (CCR5) and P-selectin glycoprotein ligand where induced by IL-12 (Bagaeva et al., 2003[11]; Rabinowich et al., 1993[158]; Deshpande et al., 2006[46]). The pathogenetic relevance of such immune cell homing has been demonstrated by the therapeutic anti-CD49d antibody natalizumab (Tysabri®), a recombinant humanized IgG4 monoclonal antibody, which blocks adhesion and homing via α4β1 and α4β7 integrin and is in successful clinical use for the treatment of MS (Nelson et al., 2018[136]; Benkert et al., 2012[21]). 

Surprisingly, clinical trials with the neutralizing anti-IL-12/23 p40 subunit antibody ustekinumab could not show the expected therapeutic benefits in MS treatment in phase II studies (Segal et al., 2008[166]). Moreover, another monoclonal anti-IL-12/23 antibody, ABT-874, demonstrated efficacy in reducing inflammatory lesions and prevention relapses, but the effect size was low compared to other agents and, hence, it was not further developed (Vollmer et al., 2011[197]). Although anti-IL23 monotherapy ameliorated EAE (Chen et al., 2006[35]), anti-p19 antibodies have so far not been tested in MS, probably due to the disappointing results observed with anti-p40 antibodies.

### Cancer

Due to its ability to activate cytotoxic cells both from the innate (NK cells) and adaptive (cytotoxic T lymphocytes) immune system, IL-12 appears to be a promising mediator of anti-tumoral immunotherapy, which has been the subject of many reviews (Tait Wojno et al., 2019[177]; Golab and Zagozdzon, 1999[63]; Lasek et al., 2014[106]; Tugues et al., 2015[188]; Yan et al., 2018[209]). Taken together, the main anti-tumoral mechanisms of IL-12 are thought to be the increase of IFN-γ production, which has anti-proliferative and pro-apoptotic effects (Castro et al., 2018[31]); the activation of proliferation and cytotoxicity of NK cells and CD4^+^ and CD8^+^ T cells; the enhancement of antibody-dependent cellular cytotoxicity (ADCC); the induction of anti-angiogenic cytokines and chemokines such as IFN-γ; the remodeling of the peritumoral extracellular matrix resulting in collapse of the tumor stroma; and the modulation of anti-tumoral immune response by influencing the major histocompatibility complex (MHC) class I molecules as well as reprogramming myeloid-derived suppressor cells (Lasek et al., 2014[106]; Campbell et al., 2015[28]). Moreover, it was found that IL-12 inhibits metastasis development in experimental tumor models in a dose-dependent way without showing signs of toxicity (Brunda et al., 1993[25]; Yue et al., 2016[216]). Interestingly, induction of IL-12 seems to mimic the natural anti-tumoral immune response and induces a very specific anti-tumor reaction (Smyth et al., 2000[172]). Consistently, neoplastic immune evasion strategies from IL-12-induced anti-tumor immunity exist, since B cells in various chronic lymphoproliferative disorders may silence the gene for IL-12Rβ2 (Il12rb2) by methylation (Airoldi et al., 2004[5]), which was associated with enhanced tumor-cell survival and proliferation *in vivo* (Airoldi et al., 2005[6]).

Additionally, it has been described that the IL-12 p40 monomer is released in higher amounts than p40 homodimer or IL-12p70 in mouse and human cancer cells. Accordingly, its serum level in patients with prostate cancer is higher compared to healthy controls. Since p40 monomer helps cancer cells to escape from cell death via internalization of IL-12Rβ1, p40 neutralization stimulated apoptosis of different cancer cells *in vitro* and *in vivo* (Kundu et al., 2017[101]). 

Possible links between IL-12 polymorphisms and various cancers have been explored in many epidemiological studies. One of the cancer-risk loci frequently identified was the SNP rs3212227 in IL12B, which shows significant association to overall cancer risk, especially among Asians, and, particularly, to hepatocellular and nasopharyngeal cancer. Additionally, the rs568408 polymorphism increases overall cancer risk among Caucasians and the risk for cervical cancer, while rs2243115 enhances the risk for brain tumors (Zheng et al., 2017[220]).

It has also been postulated that the effect of immunotherapy with monoclonal antibodies against the checkpoint receptor programmed cell death protein 1 (PD1) requires intratumoral DCs producing IL-12. In this context, the anti-PD1 antibody indirectly activates these DCs through IFN-γ released from drug-activated T cells (Garris et al., 2018[58]; Yin et al., 2016[214]).

When using IL-12 as systemic therapy, it shows toxic inflammatory responses and even lethal side effects in some cases (Ansell et al., 2002[8]; Lenzi et al., 2007[107]; Portielje et al., 1999[153]). Several studies could show that this is due to IFN-γ (Car et al., 1995[29]), but also TNF-α induction (Barrios et al., 2014[15]). To circumvent the adverse effects, different working groups established targeted transport strategies to limit the toxicity associated with systemic application. One example is a vector, in which IL-12 expression is under control of a composite promotor-containing binding motif for nuclear factor of activated T cells (NFAT), which was very effective in a murine melanoma model (Zhang et al., 2011[217]). Furthermore, gene electrotransfer, which was also used for anti-tumor *in situ* vaccination with TNF-α and IL-12 plasmid DNA in a murine melanoma model, and focused ultrasound therapy can be helpful in a more targeted treatment (Shirley et al., 2015[169]; Chen et al., 2015[34]; Kamensek et al., 2018[91]). Another approach is the delivery of IL-12 messenger RNA via lipid nanoparticles, which supressed tumorigenesis in MYC oncogene-driven hepatocellular carcinoma (Lai et al., 2018[103]). Besides, redesigning of the IL-12 molecule with deletion of the N-terminal signal peptide keeps the anti-tumor efficiency, but reduces the toxicity of the cytokine (Wang et al., 2017[204]). Moreover, the efficacy of tumor therapy with oncolytic viruses (OVs), which preferentially replicate in cancer cells and kill them while sparing healthy cells, was enhanced through viral expression of IL-12 (Nguyen et al., 2020[139]). One of these modified OVs, genetically engineered Herpes Simplex Virus-1, is used in a phase I clinical trial for the treatment of recurrent malignant glioma (NCT02062827).

Additionally, specific tumor receptors seem to be a good target for genetically modified immunotherapeutics. One example is MUC16^ecto^, which is highly expressed on most epithelial ovarian carcinomas but at low levels on normal tissues. This observation has been translated into a chimeric antigen receptor specific T cell strategy with autologous IL-12 secretion, which is investigated for the treatment of ovarian cancer (phase I trial: NCT02498912) (Koneru et al., 2015[97]).

IL-12 also seems to have great potential as adjuvant for tumor therapy especially in combination with other substances like doxorubicin (DOX), decorin (DOC) or oncolytic adenovirus expressing suicide genes, resulting in better anti-tumor immune response as shown in murine colorectal cancer, prostate cancer or 4T1 orthotopic breast cancer models (Oh et al., 2017[146]; Hu et al., 2014[82]; Ahn et al., 2016[4]; Freytag et al., 2013[53]). An IL-12-DOX-combination now in clinical evaluation is GEN-1 (EGEN-001, phase I/II trial: NCT03393884), a novel immunotherapeutic agent comprising a human IL-12-expressing plasmid encompassed within a synthetic polyethyleneglycol-polyethyleneimine-cholesterol (PPC) DNA delivery system to facilitate plasmid delivery *in vivo *and administered with pegylated liposomal doxorubicin. It showed encouraging clinical benefit and biological activity in recurrent or persistent epithelial ovarian, fallopian tube or primary peritoneal cancers (Thaker et al., 2017[180]). 

### Psoriasis

Psoriasis is a chronic inflammatory skin disease with a multifactorial etiology including genetic predisposition, environmental triggers and dysfunctions of TNF-α, dendritic cells and T cells (Griffiths and Barker, 2007[68]). Characteristic traits are infiltration of leukocytes into the skin, hyperplastic blood vessels and hyperproliferation of keratinocytes. Psoriasis vulgaris with chronic plaque formation is the most common manifestation of psoriasis (Cai et al., 2012[27]), which has long been considered to be a T_H_1-like disease. Consistently, in biopsies from psoriatic lesions high levels of IFN-γ mRNA could be detected and, furthermore, epidermal T cells produced IFN-γ (Schlaak et al., 1994[165]). Also, peripheral blood T cells from psoriasis patients were capable of producing significantly more IFN-γ compared to cells from healthy controls (Austin et al., 1999[9]). Anti-CD3/CD28 activated PBMCs, which were subsequently stimulated with IL-12 showed 233 significantly dysregulated genes after 4 h of stimulation, among these 28 IL-12-responsive genes. IL-12 stimulation was also found to significantly increase IFN-γ gene expression in anti-CD3/CD28-stimulated PBMCs and therefore might be of functional relevance for systemic inflammation in psoriasis (Enerbäck et al., 2018[50]). Furthermore, IL-12 and IL-23 levels were enhanced in lesional psoriatic skin compared to healthy and non-lesional skin (Yawalkar et al., 2009[212]). Further evidence suggests that susceptibility to psoriasis is associated with IL-12, although inter-ethnic differences exist. In an Egyptian cohort, the interaction of the single nucleotide polymorphisms rs610604 (IL-12B) and rs11209026 (IL-23R) showed a significant association with psoriasis. Moreover, the association of IL-12B with psoriasis was highly significant, whereas no association between rs20541 (IL-13) and psoriasis could be observed (Haase et al., 2015[74]). Consistently, a case-control analysis of psoriatic patients and controls from a Polish population revealed an association of IL12B rs3212227 and IL23R rs11209026 minor allele carrier status with reduced odds for psoriasis, therefore having a protective effect (Bojko et al., 2018[23]). Interestingly, it has been postulated that IL-12 together with IL-17, IL-2 and adiponectin plasma levels predicted psoriasis with a 100 % sensitivity and specificity (Cataldi et al., 2019[32]).

In experimental disease models, mice with imiquimod-induced psoriasis-like dermatitis, which were treated with an anti-IL-12/IL-23p40 monoclonal antibody (p40 mAb) showed reduced epidermal thickness and increased transepidermal water loss, as well as suppression of IL-23p19, IL-17A, IL-22 and keratin 16 gene expression, suggesting that p40 mAb not only improves dermatitis symptoms, but is also effective against skin barrier dysfunction in those mice (Takahashi et al., 2018[178]). 

However, later data showed that IL-12 receptor signaling in keratinocytes initiates a protective transcriptional program that limits skin inflammation, therefore the use of anti-p40 monoclonal antibodies and thereby collateral targeting of IL-12 might be counterproductive for psoriasis therapy (Kulig et al., 2016[100]). Consistently, the picture of psoriasis as a T_H_1-biased disease has been redrawn in the meantime, since T_H_17 cells were predominantly found in the dermis of psoriatic skin lesions (Yawalkar et al., 2009[212]; Lowes et al., 2008[112]), and IL-22 expression was also increased (Nograles et al., 2009[143]), suggesting that besides T_H_1 cells, T_H_17 and T_H_22 cells have a major influence on disease development. Thus, different T cell subsets probably contribute to plaque formation caused by immune dysregulation (Cai et al., 2012[27]). 

This matches the translational experiences made with ustekinumab. This anti-p40 antibody targeting IL-12 and IL-23 turned out to be efficient and safe in the treatment of psoriasis (Papp et al., 2008[148]; Jeon et al., 2017[88]). In accordance with the above mentioned data on genetic predisposition, IL-12-associated gene loci also seem to affect therapeutic success, since patients heterozygous (CT) for the IL12B variant (rs3213094) showed a better response to ustekinumab than the homozygous reference group (CC) (van den Reek et al., 2017[193]). Additionally, briankinumab as an additional anti-IL-12/23 antibody has been approved for the treatment of psoriasis (Gordon et al., 2012[64]). Moreover, ustekinumab has also been demonstrated to be effective in psoriatic arthritis often associated with psoriatic skin lesions. The phase III clinical trial PSUMMIT-1 (NCT01009086) resulted in more ustekinumab-treated than placebo-treated patients achieving the primary endpoint of 20 % or greater improvement in American College of Rheumatology response (ACR20) at week 24 (McInnes et al., 2013[123]). 

Further therapeutic developments in the last years have questioned the pathogenetic role of T_H_1 cells, as anti-IL-17 antibodies like secukinumab and ixekizumab demonstrated comparably efficacy to anti-IL-12/23 antibodies (Langley et al., 2014[105]). Moreover, a phase II trial revealed clinical activity of the monoclonal anti-IL23p19 antibody risankizumab that was superior to ustekinumab (Papp et al., 2017[147]). Thus, taken together, it seems that - from a clinical standpoint - T_H_17 cells seem to be more important in the pathogenesis of psoriasis than IL-12-driven T_H_1 cells. 

### Diabetes mellitus

There are two types of diabetes mellitus, type 1 (T1D) and type 2 diabetes mellitus (T2D). Characteristic for T1D is the autoimmune destruction of pancreatic islet cells. Without intervention, this leads to hyperglycemia as a result of insulin deficiency. T1D is considered to develop as a result of a reduction in immunosuppressive regulatory T cells (T_regs_), promoting the expansion of autoreactive CD4^+^ and CD8^+^ T cells (Marwaha et al., 2014[121]). On the other hand, insulin resistance, decreased insulin secretion relative to hyperglycemia, pancreatic β-cell dysfunction, disturbed renal glucose transport and incretin effects, induced by genetic and environmental risk factors are causative for T2D (Aghaei Meybodi et al., 2017[3]). Several reports provide evidence for a contribution of IL-12 to the pathogenesis of both types of diabetes mellitus. 

A frequently used murine model for T1D are nonobese diabetic (NOD) mice. Adoptive transfer of diabetogenic T_H_1 but not T_H_2 cells, as well as IL-12 administration to NOD mice actively promoted diabetes (Katz et al., 1995[93]; Trembleau et al., 1995[182]). Nevertheless, the literature shows that the pathogenesis of diabetes mellitus is more complex and an interplay of IL-12 with other molecules is involved and still not completely understood. For instance, IFN-γ production upon IL-12 stimulation is not sufficient to drive disease development, since IFN-γ knockdown induces alternative IL-12-mediated pathways. Surprisingly, IFN-γ also seems to prevent the infiltration of pancreatic β cells and the ability of APCs to activate T cells in T1D, suggesting a rather protective function of IFN-γ (Trembleau et al., 2003[183]). To the contrary, it has been shown that β-catenin accumulates in DCs of NOD mice due to hyperphosphorylation at serine 552, which is followed by activation of protein kinase Akt, driving IL-12 production and subsequent development of pathogenic IFN-γ-producing T cells (Zirnheld et al., 2019[223]). Hence, it seems that IL-12 has pathogenic as well as beneficial effects at different stages of T1D progression (Fujihira et al., 2000[54]; Nicoletti et al., 1999[140]). 

Additionally, T_H_17 cells were shown to be diabetogenic after conversion into T_H_1 cells (Mensah-Brown et al., 2006[126]; Emamaullee et al., 2009[49]; Martin-Orozco et al., 2009[120]; Bending et al., 2009[20]), leading to the assumption that T1D pathogenesis is driven by collaborative immune responses of T_H_1 cells (IL-12, IFN-γ) and T_H_17 cells (IL-23, IL-17) (Marwaha et al., 2014[121]). A study in NOD mice, which were protected from experimental autoimmune diabetes by double deficiency of the IL-17 and IFN-γ receptors, further supports this assumption (Kuriya et al., 2013[102]). 

Several studies confirmed the importance of IL-12 for human T1D. For example, neonatal levels of IL-12 were positively associated with the risk of developing T1D in childhood (Thorsen et al., 2017[181]) and children with T1D had increased IL-12 levels. Moreover, pediatric T1D patients had decreased levels of circulating T_regs_, which was negatively correlated with the abundance of IL-12 (Ryba-Stanisławowska et al., 2014[160]). Consistently, IFN-γ as well as IL-12 were found to be elevated in T1D patients without microvascular complications (MVC), the latter significantly (Shruthi et al., 2016[170]). Furthermore, a model of T1D pathogenesis has been proposed, wherein IL-12 and IL-18 synergistically enhance cytotoxic T lymphocyte and NK cell cytotoxic activity and disrupt immune regulation by T_regs_ (Dean et al., 2020[44]). 

As mentioned above, IL-12 does also play a role in the pathogenesis of T2D. A number of cytokines , including IL-6, IL-8, IL-10, IL-12 and secreted frizzled related protein 4 (SFRP4), as well as some microRNAs were described to be deregulated and associated with measures of pancreatic islet β cell function and glycemic control (Nunez Lopez et al., 2016[145]). Moreover, it has been found that the repressive histone methylation mark, H3K27me3, is decreased at the IL-12 promotor of bone marrow progenitors and passed down to wound macrophages in diet-induced obese glucose-intolerant mice. Under diabetic conditions, IL-12 production in macrophages is driven by the H3K27 demethylase Jmjd3 and can be modulated by its inhibition (Gallagher et al., 2015[57]). Furthermore, in a high-fat-diet murine model for T2D it has been shown that the disruption of IL-12 promotes angiogenesis and increases blood flow recovery (Ali et al., 2017[7]). A murine model for the treatment of T2D showed that inhibiting accumulation of group 1 innate lymphoid cells (ILC1) in the adipose tissue via IL-12-neutralizing antibodies alleviates adipose tissue fibrosis and is able to improve glycemic tolerance (Wang et al., 2019[202]). Another study revealed, that supplementation of vitamin D3 may be beneficial for T1D and T2D patients with additional autoimmune thyreoiditis. Following vitamin D3 supplementation, concentrations of inflammatory T_H_1 cytokines (IFN-γ, TNF-α, IL-2, IL-6 and IL-12) decreased, whereas levels of anti-inflammatory T_H_2-profile cytokines (IL-4, IL-5), IL-10 and IL-17 increased (Komisarenko and Bobryk, 2018[96]).

To revolutionize the treatment options for T1D, new approaches have focussed on targeting IL-12 to at least partially replace or complement the exogenous administration of insulin. As part of this, ustekinumab is investigated in new-onset T1D and following a pilot trial demonstrating safety of ustekinumab in T1D, a phase II/III trial is under way (NCT03941132). 

### Systemic lupus erythematosus

Systemic lupus erythematosus (SLE) is an autoimmune disease characterized by antibodies to double-stranded DNA that can affect various parts of the body including for instance skin, joints, kidneys or nervous system (Wang and Xia, 2019[205]). 

Several studies demonstrated that IL-12 levels are increased in SLE patients (Uzrail et al., 2019[190]; Zhou et al., 2019[222]; Guimarães et al., 2017[72]) and that risk loci for IL12RB (You et al., 2015[215]) and genetic variants of IL12B (Paradowska-Gorycka et al., 2016[149]) are associated with SLE. On the other hand, however, it has been shown that there is a T_H_17/T_reg_ imbalance in patients with SLE (Talaat et al., 2015[179]) as well as a shift in the T_H_1/T_H_2 balance towards T_H_2 cytokines (Uzrail et al., 2019[190]), suggesting that IL-12 is one cytokine among others in disease pathogenesis. 

Recently, ustekinumab has been evaluated in patients with SLE in addition to standard-of-care treatment. It resulted in better efficacy in clinical and laboratory endpoints than placebo, with a safety profile comparable with ustekinumab therapy in other diseases and might therefore be an option to improve SLE therapy (van Vollenhoven et al., 2018[194]). 

### Primary biliary cholangitis 

Primary biliary cholangitis (PBC) is an autoimmune disease which specifically affects small bile ducts of the liver, previously known as primary biliary cirrhosis. Intrahepatic small bile ducts are destroyed by lymphocyte and plasma cell infiltration and anti-mitochondrial autoantibody (AMA) as well as high serum levels of IgM are characteristic for the disease (Tsuneyama et al., 2017[187]). 

As outlined for the diseases described above, IL-12 also plays an important role for the pathogenesis of PBC. In an experimental model of PBC, using dnTGFβRII mice which have a dominant-negative transforming growth factor β receptor restricted to T cells, deletion of IL-12p35 resulted in reduced inflammation, whereas the deletion of IL-12p40 resulted in a complete protection against liver inflammation and bile duct damage (Tsuda et al., 2013[186]). Consistently, another study in mice could show that depletion of p40, leading to a decrease of the IL-12/T_H_1 as well as the IL-23/T_H_1 pathway, completely prevented the development of portal inflammatory cell infiltrates and biliary epithelial cell damage, suggesting that T_H_1 and T_H_17 effector responses affect the autoimmunity to biliary epithelial cells (Kawata et al., 2013[94]). 

Furthermore, several investigations showed that specific genetic predispositions are associated with PBC. Importantly, SNPs in the three IL-12-related genes IL12A, IL12RB2 and STAT4 are associated with PBC (Lleo et al., 2012[111]; Hirschfield et al., 2009[79]; Wasik et al., 2017[206]). Moreover, PBC T_reg_ cells were shown to be more sensitive to IL-12-induced IFN-γ expression, fostering the notion that the IL-12-IL-12Rβ2-STAT4 pathway in T_regs_ is important for disease pathogenesis and potentially treatment (Liaskou et al., 2018[108]). 

As for other IL-12-related diseases, ustekinumab was tested for the therapy of PBC patients with inadequate response to the standard therapy with ursodeoxycholic acid. Although no patient met the primary endpoint of a 40 % decline in alkaline phosphatase (ALP), the reduction of IL-17A, IFN-γ and IFN-α2 levels in patients' serum demonstrated a pharmacodynamic effect of IL-12p40 inhibition (Hirschfield et al., 2016[78]). 

### Sjögren's syndrome

Sjögren's syndrome (SS) is an autoimmune disorder resulting in dryness of mouth, eyes and other exocrine gland-connected surfaces, caused by mononuclear cell infiltrations. Further, it is associated with the production of specific autoantibodies (Jonsson et al., 2018[89]). It is classified as primary Sjögren's syndrome (pSS), when symptoms appear without associated condition, whereas secondary Sjörgen's syndrome (sSS) occurs together with another underlying autoimmune disorder, like rheumatoid arthritis, lupus erythematosus or scleroderma (Ramos-Casals et al., 2012[159]). 

Several mice models have been developed to investigate the role of IL-12 in the pathogenesis of SS. For example, McGrath-Morrow *et al.* generated a transgenic mouse model that overexpresses IL-12 in the lungs, which resulted in bronchial and alveolar abnormalities similar to those observed in Sjögren patients (McGrath-Morrow et al., 2006[122]). Moreover, in IL-12-transgenic SJL mice pilocarpine-stimulated salivary flow was significantly reduced and the number and size of lymphocytic foci was increased. IL-12 overexpression in CBA mice led to mononuclear infiltration of salivary and lacrimal glands, expansion of bronchial lymphoid tissue and decreased mucociliary clearance reminiscent of SS (Vosters et al., 2009[199]). Besides, IL-12 mRNA was predominantly expressed in the proinflammatory stage of autoimmune sialadenitis in MRL/lpr mice with experimental SS (Yanagi et al., 1996[210]) and plasma IL-12 was significantly increased in SS-like NOD mice, while anti-IL-12 alleviated the SS-like symptoms (Qi et al., 2019[157]). 

Furthermore, it has been shown that the IL12A rs485497 polymorphism is associated with pSS and that IL-12p70 serum levels in patients with active disease are higher than in control subjects, whereas serum IL-35 levels were associated with low disease activity, indicating an involvement of the IL-12/IL-35 balance in the pathogenesis of pSS (Fogel et al., 2018[52]). Another study revealed increased serum concentrations of IL-10 and IL-12 in pSS patients, which were both significantly correlated with pro-inflammatory IL-6. Additionally, the T_H_1/T_H_2 ratio was significantly decreased in those patients (Girón-González et al., 2009[62]). Moreover, it has been shown that IL-12p40 and IL-15 levels were significantly decreased, while IL-1β and TNF-α were significantly elevated in the plasma of SS patients. In addition, significant differences in IL-12p40 were described between patients with or without extra-glandular manifestations (Szodoray et al., 2004[176]) and high expression of IL-12 by mononuclear cell infiltrates in minor salivary glands has been observed (Manoussakis et al., 2007[119]). Interestingly, mesenchymal stem cell transplantation (MSCT), an experimental therapeutic strategy proposed for SS, downregulated T_H_17 and T_FH_ cells, but upregulated T_regs_ and reduced IL-12 production in SS patients as well as in mice, indicating that MSCs improve SS by suppressing the production of IL-12 in DCs (Shi et al., 2018[168]). Taken together, IL-12 might be a potential therapeutic target for SS that deserves further research.

### Rheumatoid arthritis

Rheumatoid arthritis (RA) is caused by genetic as well as environmental factors. Characteristics include synovial inflammation and swelling possibly leading to skeletal deformation caused by destruction of cartilage and bones (McInnes and Schett, 2011[124]). As for the other conditions discussed, RA manifestation is influenced by T_H_1 and T_H_17 cells, associated with IL-12 and IL-23 (Cornelissen et al., 2009[41]).

Collagen-induced arthritis (CIA) is a common mouse model for RA. The incidence and severity of CIA is significantly reduced in IL-12p40-deficient mice (McIntyre et al., 1996[125]). Similarly, treatment of type II collagen immunized DBA/1 mice with a neutralizing anti-IL-12 (p40) antibody mitigated the clinical and histopathological disease severity extensively (Malfait et al., 1998[117]). However, later studies dissecting the role of IL-12 and IL-23 rather provided evidence for a protective function of IL-12 by showing that mice specifically lacking IL-23 (p19^-/-^) were protected from developing clinical signs of disease, whereas a specific lack of IL-12 (p35^-/-^) resulted in aggravated collagen-induced arthritis (Murphy et al., 2003[130]). Consistently, IL-12 administration at the time of arthritis onset had a stimulatory effect on disease activity, while it seems to be suppressive in established CIA (Joosten et al., 1997[90]). Nevertheless, IL-12 does not remain uninvolved. Several gene polymorphism studies showed that IL-12B gene polymorphisms have an impact on RA pathogenesis, since, for instance, investigations in the Polish population showed that frequencies of the rs3212227 CC of the IL12B gene were statistically higher in RA patients compared to controls and that the IL12B 1188A/C allele as well as IL-12p70 protein levels are likely to be associated with RA (Paradowska-Gorycka et al., 2017[150]). Further, in a Bulgarian population, the IL12B rs17860508 polymorphism was associated with RA and RA patients with rs3212227 AA genotype of IL12B showed increased serum levels of IL-12p40 and IL-23 (Manolova et al., 2020[118]). By contrast, a meta-analysis of 17 case-control studies demonstrated that IL-12B rs3212227 and rs6887695 polymorphisms do not confer susceptibility to RA (Yang et al., 2017[211]). However, compared with healthy controls higher IL-12 levels can be found in the synovia (Morita et al., 1998[128]) and serum (Paradowska-Gorycka et al., 2017[150]; Cordero et al., 2001[40]) of RA patients. Quite a number of drugs are used for the treatment of RA and some of their mechanisms have been linked with IL-12. For example, it has been suggested that methotrexate induces a down-regulation of IL-12 (Hobl et al., 2011[80]) and modulates the T_H_1/T_H_2 balance towards a T_H_2 profile by induction of IL-10 secretion and reduction of IL-12R and C-X-C motif chemokine receptor 3 (CXCR3) (Herman et al., 2008[76]). Moreover, the phosphodiesterase 4 (PDE4) inhibitor Apremilast (Otezla^®^) strongly inhibited IL-12/IL-23p40 in cultured synovial fluid mononuclear cells from patients with active RA, psoriatic arthritis or peripheral spondyloarthritis (Kragstrup et al., 2019[98]). Another PDE4 inhibitor, Ibudilast, reduced the expression and/or secretion of TNF and IL-12/IL-23p40 in activated human leukocytes and RA synovial fibroblasts and further inhibited T_H_17 cell responses *in vivo* (Clanchy and Williams, 2019[37]). Sinomenine, an alkaloid extracted from the Chinese medical plant *Sinomenium acutum*, which is approved in China, suppressed RA progression by regulating the secretion of several inflammatory cytokines, such as IL-12p40, IL-6, TNF-α (Liu et al., 2018[110]). However, therapeutic strategies specifically targeting IL-12 have not been considered so far, since the role of IL-12 in RA does not seem to be essential.

## Concluding Remarks

The history of IL-12 is marked by ups and downs. While it was associated with lots of pathological conditions shortly after its discovery based on methods and strategies detecting or targeting the p40 subunit, the diversification of the IL-12 family led to re-attribution of many of the functions initially assigned to IL-12 and other members like IL-23 gained more attention.

However, it must not be forgotten that targeting IL-12 together with IL-23 has become an established therapeutic strategy in IBD, psoriasis and peripheral spondylo-arthritis, while experiences with anti-p19 antibodies are still limited. Thus, IL-12 is a key cytokine to consider in these diseases. Moreover, there are areas, where it seems to be “rediscovered”, particularly in the cancer field, where several promising approaches related to IL-12 are currently investigated.

Taken together, this cytokine positioned at an important nexus between the innate and adaptive response displays crucial functions in health and disease that are translationally relevant. Despite being known for more than 30 years now, we are still far away from completely understanding its involvement in physiological and pathological processes.

## Notes

Karen A.-M. Ullrich and Lisa Lou Schulze contributed equally as first authors.

## Competing interests

None.

## Funding

The research of MFN and SZ was supported by the Interdisciplinary Center for Clinical Research (IZKF) and the ELAN program of the University Erlangen-Nuremberg, the Else Kröner-Fresenius-Stiftung, the Fritz Bender-Stiftung, the Dr Robert Pfleger Stiftung, the Litwin IBD Pioneers Initiative of the Crohn's and Colitis Foundation of America (CCFA), the Kenneth Rainin Foundation, the Ernst Jung-Stiftung for Science and Research, the German Crohn's and Colitis Foundation (DCCV) and the German Research Foundation (DFG) through individual grants (ZU 377/4-1) and the Collaborative Research Centers TRR241, 643, 796 and 1181.
